# Crystal structure of the possible sulindac impurity 2-(5-fluoro-2-methyl-1*H*-inden-3-yl)aceto­nitrile

**DOI:** 10.1107/S2056989025002622

**Published:** 2025-03-27

**Authors:** Wu Yun-Deng, Wan Hui, Xia Yun, Ni Jie, Li Jian, Zhang Hui, Xu Xiang-Yang, Xie Jun

**Affiliations:** a Technique Center, Jinling Pharmaceutical Company Limited, 58 Xingang Road Qixia, district, Nanjing, Jiangsu 210046, People’s Republic of China; Vienna University of Technology, Austria

**Keywords:** sulindac, impurity, 2-(5-fluoro-2-methyl-1*H*-inden-3-yl)aceto­nitrile, synthesis, crystal structure

## Abstract

The title compound,C_12_H_10_FN, was identified as a possible critical degradation impurity of sulindac, a therapeutic COX-2 inhibitor for rheumatoid arthritis. Single-crystal X-ray analysis revealed two conformationally slightly different mol­ecules in the asymmetric unit (*Z*′ = 2), each containing an indene ring system. In the crystal structure, [100] chains formed through C—H⋯N inter­actions are connected into a tri-periodic supra­molecular structure by further C—H⋯F and C—H⋯π inter­actions through *P*2_1_/*n* symmetry operations.

## Abstract 2

The asymmetric unit of the title compound consists of two mol­ecules with nearly identical conformations. The angles between the cyanide group and the corresponding indene ring plane are 64.09 (16) and 64.72 (14)°.

## 1. Chemical context

Sulindac {systematic name [(*Z*)-2-methyl-1-[(4-methyl­sulf­in­yl­phen­yl)methyl­ene]-5-fluoro-1*H*-inden-3-acetic acid]} is a nonsteroidal anti-inflammatory drug (NSAID) that exhibits selective cyclo­oxygenase-2 (COX-2) inhibitory activity, effectively suppressing COX-2 overexpression through competitive inhibition. It is applied for clinical management of rheumatoid arthritis and degenerative joint disorders (Boolbol, 1996 ▸).

While impurity profiling represents a critical component of pharmaceutical development, the present investigation focuses on the characterization of a key process-related impurity compound in sulindac synthesis, namely 2-(5-fluoro-2-methyl-1*H*-inden-3-yl)aceto­nitrile (**1**). Based on single-crystal X-ray diffraction analysis, we have now unambiguously determined its configuration, representing novel structural data in pharmaceutical crystallography and report the results here.
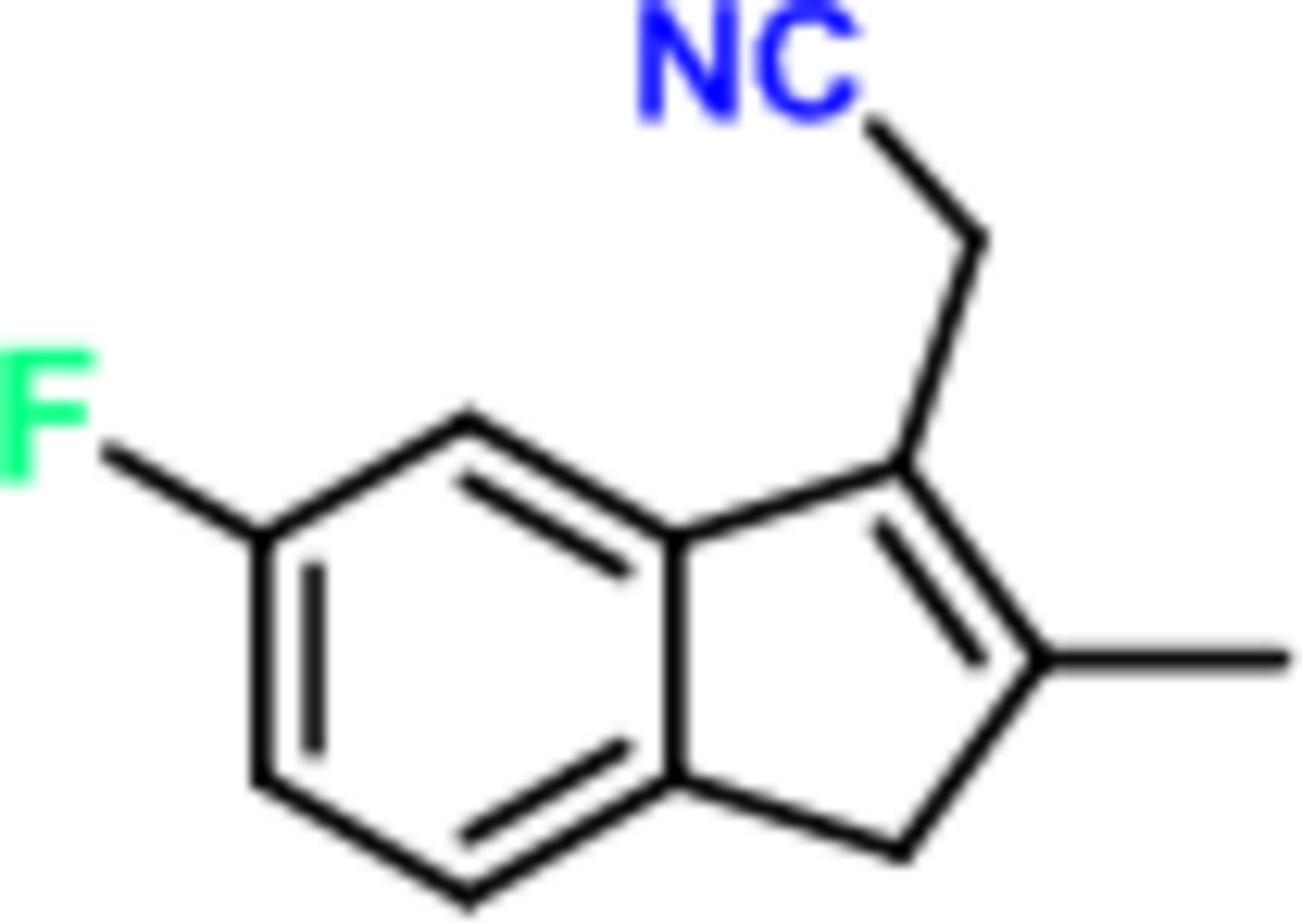


## 2. Structural commentary

**Figure 1:**
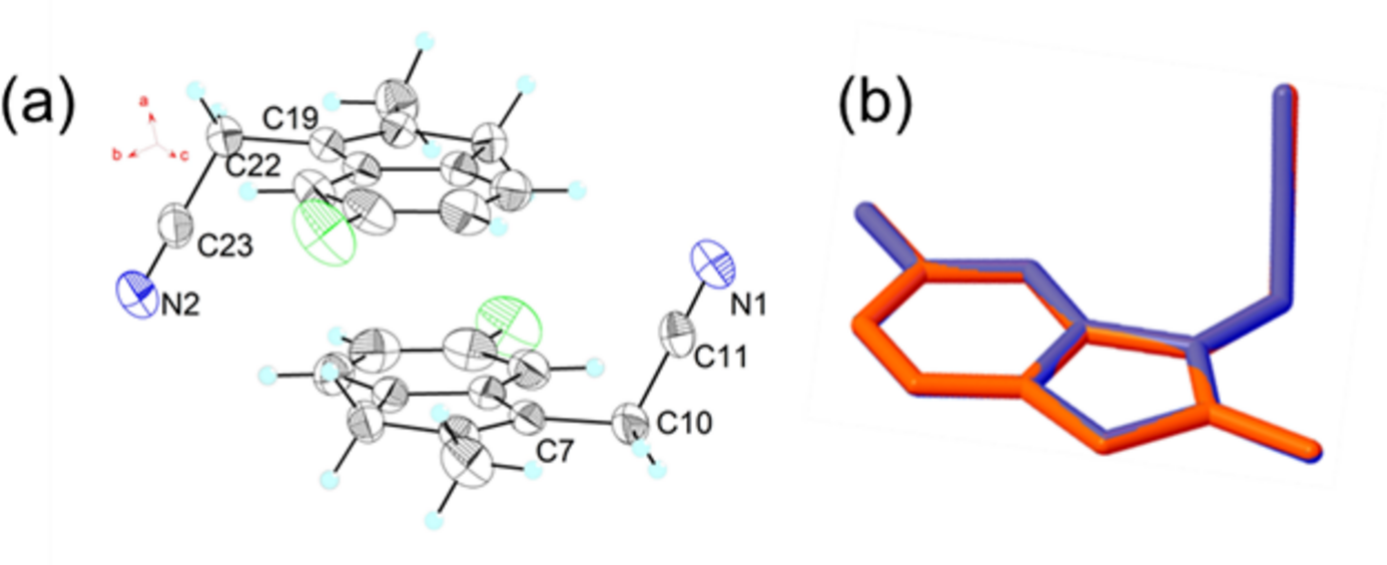
(*a*) The asymmetric unit of (**1**) with displacement ellipsoids drawn at the 50% probability level; (*b*) overlay plot of the two independent mol­ecules.

**Figure 2:**
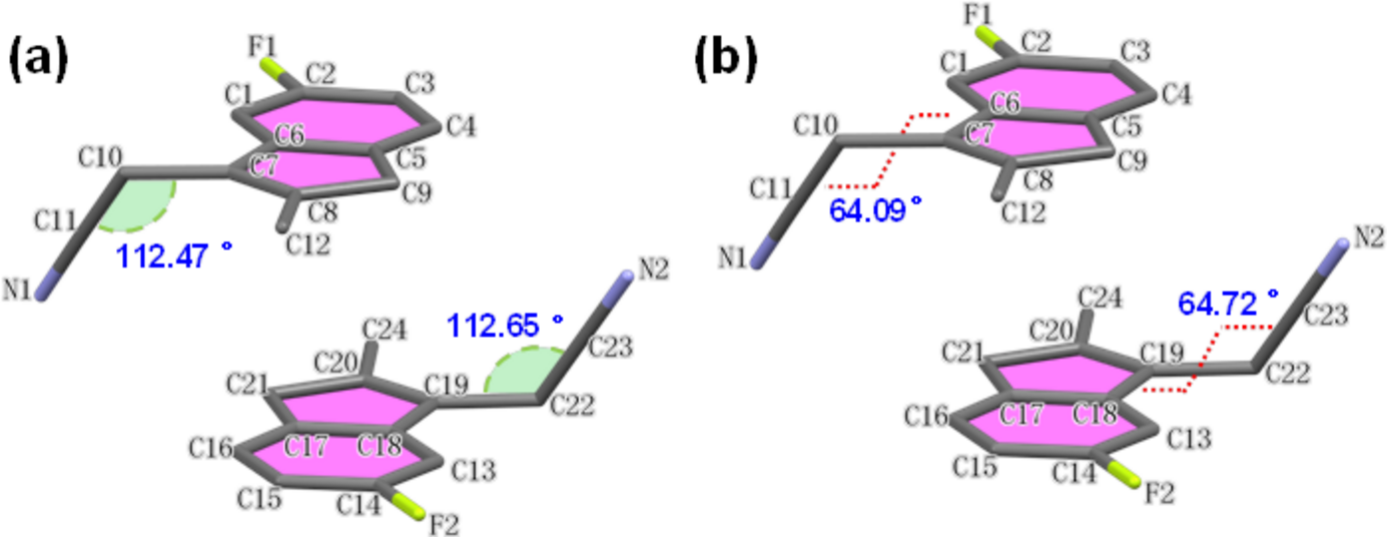
(*a*) The angle between the cyanide group and adjacent atoms in each of the two independent mol­ecules; (*b*) the cyanide group and its dihedral angle with the corresponding indene ring plane in each of the two independent mol­ecules.

The asymmetric unit of (**1**) comprises two mol­ecules and is illustrated in Fig. 1 ▸*a*; an overlay plot of the two mol­ecules (one of which is inverted relative to the other) is shown in Fig. 1 ▸*b*. The root-mean-square-deviation between the two mol­ecules is only 0.009 Å, with *d*_max_ of 0.020 Å between N1 and N2. The angles C7—C10—C11 and C19—C22—C23 are 112.47 (10) and 112.65 (10)°, respectively (Fig. 2 ▸*a*), and the torsion angles between the cyanide group and its corresponding indene ring plane is 64.09 (16) and 64.72 (14)° in the two independent mol­ecules (Fig. 2 ▸*b*). The bond lengths in the two mol­ecules are all within normal range.

## 3. Supra­molecular features

**Figure 3:**
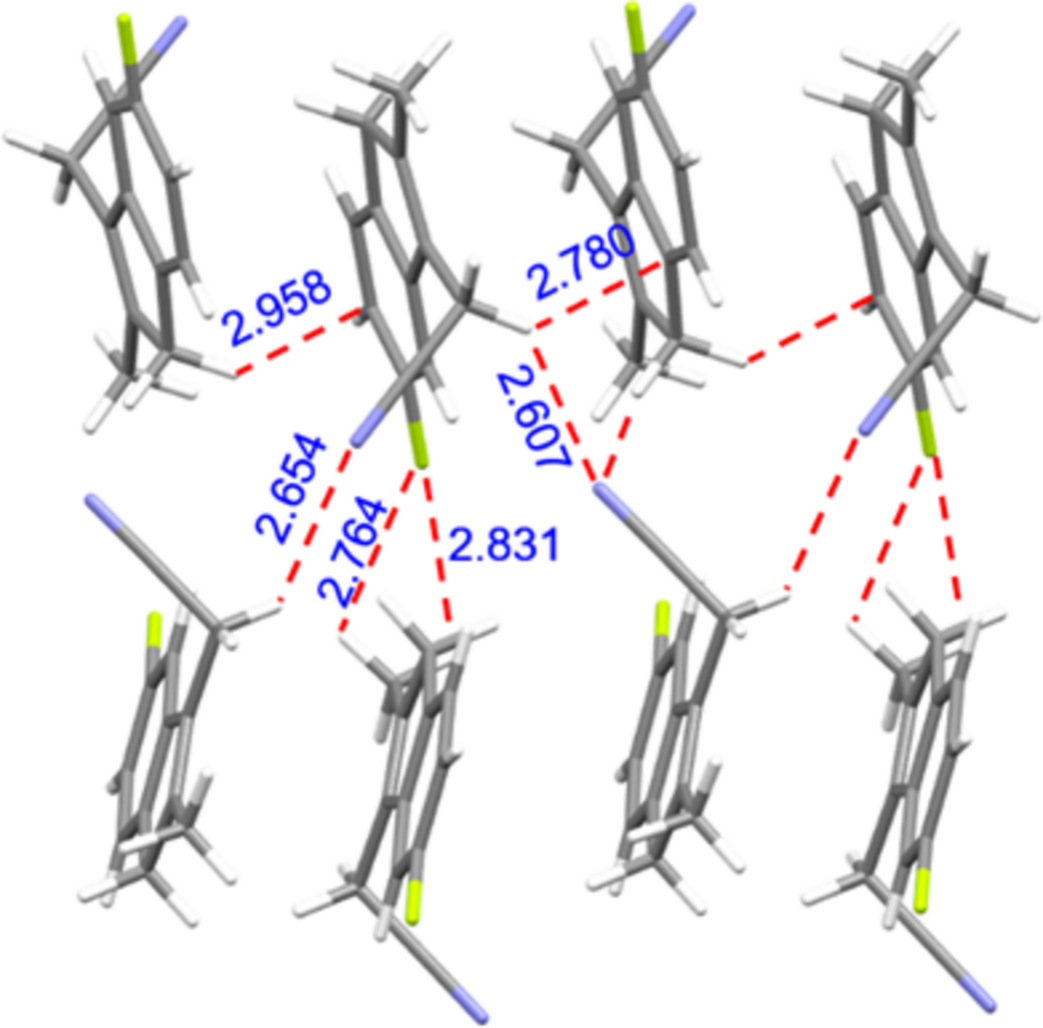
Inter­molecular inter­actions (C—H⋯N, C—H⋯F and C—H⋯π) between adjacent mol­ecules shown as red dashed lines. Color codes: C (gray), N (blue), F(green) and H (white).

**Figure 4:**
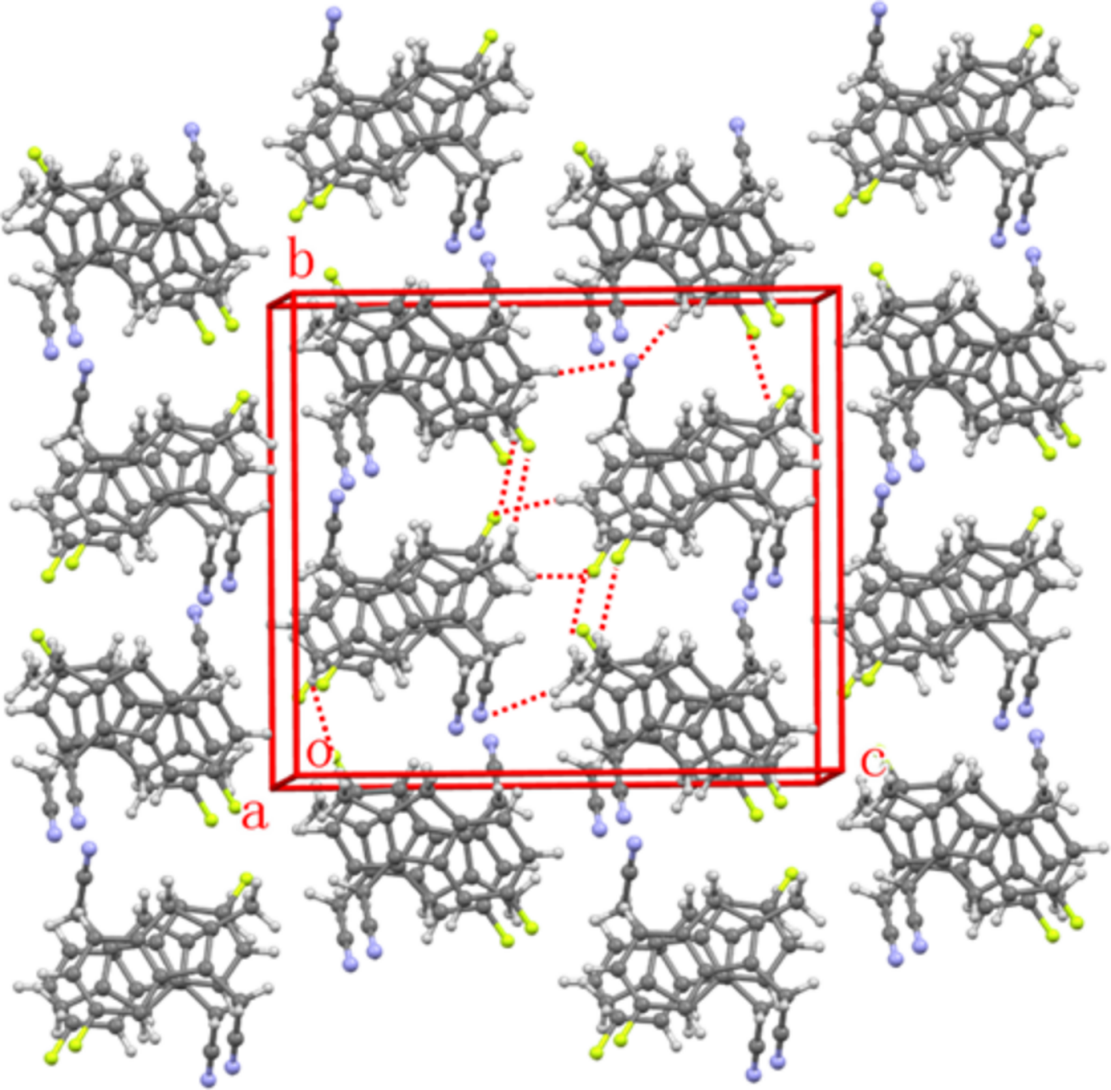
Packing plot of (**1**) approximately along [100]. C—H⋯N and C—H⋯F inter­actions are shown as red dotted lines.

As shown in Fig. 3 ▸, the methyl­ene groups (C10, C22) attached to the —C≡N moiety act as hydrogen-bond donors to the cyanide N atoms (N2, N1) of adjacent mol­ecules. These inter­actions (Table 1 ▸) link the mol­ecules into an infinite supra­molecular chain extending parallel to [100]. Other inter­actions shown in Fig. 3 ▸ include C—H⋯F inter­actions (Table 1 ▸) as well as C—H⋯π inter­actions [C22–H22*A*⋯π (2.780 Å) and C12—H12*B*⋯π (2.958 Å)], which connect the mol­ecules into a tri-periodic supra­molecular structure. A packing plot of (**1**) is shown in Fig. 4 ▸.

## 4. Database survey

A search of the Cambridge Structural Database (CSD, version 2024.1.0; Groom *et al.*, 2016 ▸) was conducted using the keyword methyl­indene, which retrieved eleven relevant entries: BANNUA (Tsuno *et al.*, 2003 ▸), CIRMIA (Santi *et al.*, 2007 ▸), DOBVIZ (Biali & Rappoport, 1986 ▸), FUNPAG (Xu *et al.*, 2010 ▸), HEXRAD (Bonifaci *et al.*, 1994 ▸), ICEXOD (Halterman *et al.*, 2000 ▸), SUZYER (Stenzel *et al.*, 2001 ▸), MIJKIZ (Enders *et al.*, 2002 ▸), NOYTOK (Brase *et al.*, 1998 ▸), XAWFEG (Shapiro *et al.*, 1999 ▸), and RESZEU (Herrmann *et al.*, 1997 ▸). The primary distinction among these compounds lies in the substitution patterns of the methyl group on the indene ring. Notably, an indene derivative bearing both fluorine and cyano substituents has been obtained and reported exclusively in the present work. This comparative analysis underscores the structural novelty of the title compound, particularly its unique combination of hydrogen-bonding patterns (C—H⋯N and C—H⋯F).

## 5. Synthesis and crystallization

**Figure 5:**
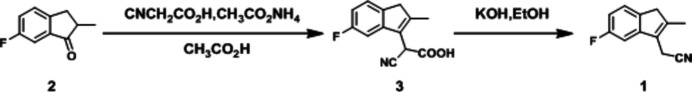
Synthesis scheme of the title compound (**1**).

Compound (**1**) was prepared according to a literature method (Xu *et al.*, 2020 ▸; Dai *et al.*, 2009 ▸). The preparation procedure is schematically shown in Fig. 5 ▸. A 100 ml round-bottomed flask equipped with a magnetic stirring bar was charged with a mixture of 6-fluoro-2-methyl-2,3-di­hydro-1*H*-inden-1-one, (**2**), (20.35 g, 124 mmol), cyano­acetic acid (13.65 g, 160.4 mmol), acetic acid (7.8 g, 129.9 mmol) and ammonium acetate (3.1 g, 40.2 mmol) in toluene (50 ml). The mixture was refluxed for 24 h and then cooled to room temperature. The solvent was removed *in vacuo*, and the residue (**3**) was dissolved in ethanol without further purification. The resulting solution was added to a potassium hydroxide solution (165 g, 25%_wt_, *w*/*w*, 735.2 mmol) and heated to reflux for 13 h. Then, the ethanol was removed *in vacuo*, followed by the addition of 300 ml of water. The pH value was adjusted to 8 using concentrated hydro­chloric acid, and the mixture was then extracted with di­chloro­methane (150 ml). The aqueous layer was collected and further adjusted to a pH value of 2 with concentrated hydro­chloric acid, resulting in the precipitation of a significant amount of a yellow solid of (**1**). The solid was then filtered off, washed with water, and dried in air. Yield: 17.25 g, 85.2%. ^1^H NMR spectrum (Varian Unity Inova 500 MHz, DMSO-*d*_6_, ppm): δ: 7.38 (*dd*, 1H, *J*_1_ = 5.55 Hz, *J*_2_ = 7.85 Hz), 7.23 (*dd*, 1H, *J*_1_ = 2.2 Hz, *J*_2_ = 9.5 Hz), 6.94 (*m*, 1H), 3.87 (*s*, 2H), 3.35 (*s*, 2H), 2.13 (*s*, 3H). Single crystals were obtained by slow evaporation of a saturated solution of (**1**) in a di­chloro­methane–ethanol mixture (4:1, *v*/*v*) at room temperature over 20 d.

## 6. Refinement

Crystal data, data collection and structure refinement details are summarized in Table 2 ▸. Hydrogen atoms attached to carbon atoms were placed in calculated positions and constrained with AFIX instructions.

## 7. Authorship contribution statement

The submitted manuscript includes contributions from eight authors. Yun-Deng Wu conceptualized and designed the research framework, conducted comprehensive data analysis, and drafted the manuscript. Yun-Deng Wu, Hui Wan, Yun Xia and Jie Ni performed the synthesis, isolation, purification, and characterization of the title compound. Jian Li, Hui Zhang, Xiangyang Xu and Jun Xie contributed to the single-crystal cultivation and associated experimental evaluations. All co-authors participated in the critical revision and final approval of the manuscript for publication.

## Supplementary Material

Crystal structure: contains datablock(s) I. DOI: 10.1107/S2056989025002622/wm5750sup1.cif

Structure factors: contains datablock(s) I. DOI: 10.1107/S2056989025002622/wm5750Isup2.hkl

Supporting information file. DOI: 10.1107/S2056989025002622/wm5750Isup3.cml

CCDC reference: 2219596

Additional supporting information:  crystallographic information; 3D view; checkCIF report

Additional supporting information:  crystallographic information; 3D view; checkCIF report

## Figures and Tables

**Table 1:** Hydrogen-bond geometry (Å, °)

*D*—H⋯*A*	*D*—H	H⋯*A*	*D*⋯*A*	*D*—H⋯*A*
C10—H10*B*⋯N2^i^	0.99	2.65	3.3978 (18)	132
C22—H22*A*⋯N1^ii^	0.99	2.61	3.4010 (16)	137
C24—H24*C*⋯F2^iii^	0.98	2.76	3.227 (2)	110
C24—H24*B*⋯F2^iii^	0.98	2.83	3.227 (2)	105

Symmetry codes: (i) 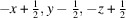; (ii) 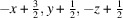; (iii) 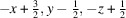.

**Table 2:** Experimental details

Crystal data	
Chemical formula	C_12_H_10_FN
*M*_r_	187.21
Crystal system, space group	Monoclinic, *P*2_1_/*n*
Temperature (K)	170
*a*, *b*, *c* (Å)	7.5248 (5), 15.1897 (10), 17.1823 (9)
β (°)	99.176 (2)
*V* (Å^3^)	1938.8 (2)
*Z*	8
Radiation type	Mo *K*α
μ (mm^−1^)	0.09
Crystal size (mm)	0.42 × 0.29 × 0.23

Data collection	
Diffractometer	Bruker *SMARTAPEX* CCD area detector
Absorption correction	Multi-scan (*SADABS*; Krause *et al.*, 2015 ▸)
*T*_min_, *T*_max_	0.654, 0.745
No. of measured, independent and observed [*I* > 2σ(*I*)] reflections	28954, 4137, 3530
*R*_int_	0.031
(sin θ/λ)_max_ (Å^−1^)	0.634

Refinement	
*R*[*F*^2^ > 2σ(*F*^2^)], *wR*(*F*^2^), *S*	0.039, 0.105, 1.03
No. of reflections	4137
No. of parameters	256
H-atom treatment	H-atom parameters constrained
Δρ_max_, Δρ_min_ (e Å^−3^)	0.21, −0.21

Computer programs: *APEX3* and *SAINT* (Bruker, 2016 ▸), *SHELXT* (Sheldrick, 2015*a* ▸), *SHELXL* (Sheldrick, 2015*b* ▸), *OLEX2* (Dolomanov *et al.*, 2009 ▸) and *publCIF* (Westrip, 2010 ▸).

## supplementary crystallographic information

### 2-(5-Fluoro-2-methyl-1H-inden-3-yl)acetonitrile Crystal data

**Table:**

C_12_H_10_FN	*F*(000) = 784
*M**_r_* = 187.21	*D*_x_ = 1.283 Mg m^−^^3^
Monoclinic, *P*2_1_/*n*	Mo *K*α radiation, λ = 0.71073 Å
*a* = 7.5248 (5) Å	Cell parameters from 9932 reflections
*b* = 15.1897 (10) Å	θ = 2.7–26.7°
*c* = 17.1823 (9) Å	µ = 0.09 mm^−^^1^
β = 99.176 (2)°	*T* = 170 K
*V* = 1938.8 (2) Å^3^	Block, colourless
*Z* = 8	0.42 × 0.29 × 0.23 mm

### 2-(5-Fluoro-2-methyl-1H-inden-3-yl)acetonitrile Data collection

**Table:**

Bruker SMART APEX CCD area detector diffractometer	4137 independent reflections
Radiation source: sealed tube	3530 reflections with *I* > 2σ(*I*)
Graphite monochromator	*R*_int_ = 0.031
Detector resolution: 8 pixels mm^-1^	θ_max_ = 26.8°, θ_min_ = 2.4°
ω and φ scans	*h* = −9→9
Absorption correction: multi-scan (SADABS; Krause *et al.*, 2015)	*k* = −19→19
*T*_min_ = 0.654, *T*_max_ = 0.745	*l* = −21→21
28954 measured reflections	

### 2-(5-Fluoro-2-methyl-1H-inden-3-yl)acetonitrile Refinement

**Table:**

Refinement on F^2^	Hydrogen site location: inferred from neighbouring sites
Least-squares matrix: full	H-atom parameters constrained
R[F^2^ > 2σ(F^2^)] = 0.039	w = 1/[σ^2^(F_o_^2^) + (0.0464P)^2^ + 0.5292P] where P = (F_o_^2^ + 2F_c_^2^)/3
wR(F^2^) = 0.105	(Δ/σ)_max_ = 0.001
S = 1.03	Δρ_max_ = 0.21 e Å^−^^3^
4137 reflections	Δρ_min_ = −0.21 e Å^−^^3^
256 parameters	Extinction correction: SHELXL (Sheldrick, 2015b), Fc^*^=kFc[1+0.001xFc^2^λ^3^/sin(2θ)]^-1/4^
0 restraints	Extinction coefficient: 0.0048 (7)
Primary atom site location: dual	

### 2-(5-Fluoro-2-methyl-1H-inden-3-yl)acetonitrile Special details

**Table:**

Geometry. All esds (except the esd in the dihedral angle between two l.s. planes) are estimated using the full covariance matrix. The cell esds are taken into account individually in the estimation of esds in distances, angles and torsion angles; correlations between esds in cell parameters are only used when they are defined by crystal symmetry. An approximate (isotropic) treatment of cell esds is used for estimating esds involving l.s. planes.

### 2-(5-Fluoro-2-methyl-1H-inden-3-yl)acetonitrile Fractional atomic coordinates and isotropic or equivalent isotropic displacement parameters (Å^2^)

**Table:**

	*x*	*y*	*z*	*U*_iso_*/*U*_eq_	
F1	0.08006 (14)	0.18981 (6)	0.05253 (6)	0.0683 (3)	
N1	0.46276 (16)	0.13422 (7)	0.35764 (7)	0.0451 (3)	
C1	0.16171 (16)	0.24962 (8)	0.17953 (8)	0.0373 (3)	
H1	0.142597	0.194447	0.202895	0.045*	
C2	0.13519 (18)	0.26100 (9)	0.09871 (8)	0.0446 (3)	
C3	0.16163 (18)	0.33907 (10)	0.06189 (8)	0.0468 (3)	
H3	0.141755	0.343036	0.006001	0.056*	
C4	0.21793 (17)	0.41208 (9)	0.10788 (7)	0.0403 (3)	
H4	0.236765	0.466889	0.083839	0.048*	
C5	0.24625 (15)	0.40411 (8)	0.18899 (7)	0.0314 (3)	
C6	0.21782 (14)	0.32325 (7)	0.22468 (7)	0.0296 (2)	
C7	0.25771 (15)	0.33531 (8)	0.31024 (7)	0.0308 (3)	
C8	0.30813 (16)	0.41860 (8)	0.32741 (7)	0.0343 (3)	
C9	0.30565 (16)	0.47028 (8)	0.25250 (7)	0.0356 (3)	
H9A	0.219788	0.519996	0.249653	0.043*	
H9B	0.426803	0.493474	0.248492	0.043*	
C10	0.23961 (19)	0.26298 (8)	0.36817 (8)	0.0410 (3)	
H10A	0.262345	0.287413	0.422263	0.049*	
H10B	0.114754	0.240222	0.358457	0.049*	
C11	0.36465 (16)	0.19013 (8)	0.36265 (7)	0.0340 (3)	
C12	0.3611 (2)	0.46070 (10)	0.40633 (8)	0.0503 (4)	
H12A	0.282934	0.511371	0.411000	0.075*	
H12B	0.486549	0.480414	0.411934	0.075*	
H12C	0.348725	0.417899	0.447807	0.075*	
F2	0.92640 (14)	0.55350 (6)	0.40592 (5)	0.0692 (3)	
N2	0.52928 (17)	0.59111 (8)	0.10051 (8)	0.0555 (3)	
C13	0.83947 (16)	0.48567 (8)	0.28300 (7)	0.0366 (3)	
H13	0.857248	0.539097	0.256548	0.044*	
C14	0.86929 (19)	0.47951 (9)	0.36423 (8)	0.0450 (3)	
C15	0.84553 (19)	0.40399 (10)	0.40560 (8)	0.0465 (3)	
H15	0.868240	0.403684	0.461595	0.056*	
C16	0.78762 (17)	0.32816 (9)	0.36380 (7)	0.0394 (3)	
H16	0.769749	0.275137	0.390833	0.047*	
C17	0.75646 (15)	0.33098 (7)	0.28246 (7)	0.0310 (3)	
C18	0.78197 (14)	0.40920 (7)	0.24219 (7)	0.0292 (2)	
C19	0.73809 (14)	0.39234 (7)	0.15742 (6)	0.0280 (2)	
C20	0.68786 (15)	0.30797 (7)	0.14500 (7)	0.0302 (2)	
C21	0.69555 (16)	0.26085 (7)	0.22262 (7)	0.0328 (3)	
H21A	0.575673	0.237782	0.229008	0.039*	
H21B	0.782483	0.211482	0.227027	0.039*	
C22	0.75293 (16)	0.46131 (7)	0.09589 (7)	0.0336 (3)	
H22A	0.877380	0.484612	0.103829	0.040*	
H22B	0.729198	0.433713	0.043056	0.040*	
C23	0.62711 (17)	0.53441 (8)	0.09855 (7)	0.0370 (3)	
C24	0.63087 (19)	0.26147 (8)	0.06865 (8)	0.0416 (3)	
H24A	0.626613	0.303465	0.025088	0.062*	
H24B	0.511239	0.235723	0.068025	0.062*	
H24C	0.717319	0.214674	0.062657	0.062*	

### 2-(5-Fluoro-2-methyl-1H-inden-3-yl)acetonitrile Atomic displacement parameters (Å^2^)

**Table:**

	*U*^11^	*U*^22^	*U*^33^	*U*^12^	*U*^13^	*U*^23^
F1	0.0791 (7)	0.0627 (6)	0.0581 (6)	0.0011 (5)	−0.0044 (5)	−0.0283 (5)
N1	0.0494 (6)	0.0335 (6)	0.0496 (7)	0.0066 (5)	−0.0003 (5)	0.0027 (5)
C1	0.0356 (6)	0.0331 (6)	0.0431 (7)	0.0031 (5)	0.0060 (5)	−0.0015 (5)
C2	0.0405 (7)	0.0498 (8)	0.0415 (7)	0.0044 (6)	0.0007 (5)	−0.0147 (6)
C3	0.0435 (7)	0.0669 (9)	0.0291 (6)	0.0092 (6)	0.0035 (5)	0.0015 (6)
C4	0.0367 (6)	0.0489 (7)	0.0356 (6)	0.0039 (5)	0.0066 (5)	0.0114 (5)
C5	0.0265 (5)	0.0343 (6)	0.0335 (6)	0.0033 (4)	0.0055 (4)	0.0064 (5)
C6	0.0259 (5)	0.0309 (6)	0.0323 (6)	0.0046 (4)	0.0059 (4)	0.0026 (4)
C7	0.0315 (6)	0.0314 (6)	0.0306 (6)	0.0080 (4)	0.0078 (4)	0.0047 (4)
C8	0.0336 (6)	0.0342 (6)	0.0342 (6)	0.0071 (5)	0.0025 (5)	0.0010 (5)
C9	0.0348 (6)	0.0300 (6)	0.0410 (7)	0.0001 (5)	0.0034 (5)	0.0050 (5)
C10	0.0522 (8)	0.0365 (7)	0.0376 (7)	0.0112 (6)	0.0172 (6)	0.0102 (5)
C11	0.0418 (6)	0.0298 (6)	0.0284 (6)	−0.0004 (5)	−0.0001 (5)	0.0046 (4)
C12	0.0586 (9)	0.0478 (8)	0.0411 (7)	0.0075 (7)	−0.0023 (6)	−0.0091 (6)
F2	0.0937 (7)	0.0482 (5)	0.0558 (5)	0.0070 (5)	−0.0178 (5)	−0.0238 (4)
N2	0.0527 (7)	0.0394 (6)	0.0758 (9)	0.0120 (6)	0.0146 (6)	0.0182 (6)
C13	0.0369 (6)	0.0294 (6)	0.0410 (7)	0.0045 (5)	−0.0011 (5)	−0.0025 (5)
C14	0.0485 (8)	0.0396 (7)	0.0426 (7)	0.0086 (6)	−0.0061 (6)	−0.0145 (6)
C15	0.0524 (8)	0.0559 (8)	0.0294 (6)	0.0127 (6)	0.0012 (6)	−0.0034 (6)
C16	0.0413 (7)	0.0441 (7)	0.0334 (6)	0.0068 (5)	0.0079 (5)	0.0059 (5)
C17	0.0291 (5)	0.0316 (6)	0.0330 (6)	0.0046 (4)	0.0067 (4)	0.0021 (5)
C18	0.0257 (5)	0.0289 (6)	0.0325 (6)	0.0048 (4)	0.0033 (4)	0.0001 (4)
C19	0.0267 (5)	0.0268 (5)	0.0305 (6)	0.0035 (4)	0.0051 (4)	0.0015 (4)
C20	0.0302 (5)	0.0282 (6)	0.0328 (6)	0.0022 (4)	0.0066 (4)	0.0000 (4)
C21	0.0356 (6)	0.0273 (6)	0.0363 (6)	−0.0003 (4)	0.0083 (5)	0.0032 (5)
C22	0.0369 (6)	0.0295 (6)	0.0349 (6)	0.0014 (5)	0.0069 (5)	0.0043 (5)
C23	0.0374 (6)	0.0312 (6)	0.0416 (7)	−0.0018 (5)	0.0040 (5)	0.0100 (5)
C24	0.0536 (8)	0.0328 (6)	0.0378 (7)	0.0006 (5)	0.0052 (6)	−0.0049 (5)

### 2-(5-Fluoro-2-methyl-1H-inden-3-yl)acetonitrile Geometric parameters (Å, º)

**Table:**

F1—C2	1.3660 (15)	F2—C14	1.3648 (15)
N1—C11	1.1375 (16)	N2—C23	1.1370 (17)
C1—H1	0.9500	C13—H13	0.9500
C1—C2	1.3820 (19)	C13—C14	1.3810 (18)
C1—C6	1.3880 (17)	C13—C18	1.3898 (16)
C2—C3	1.373 (2)	C14—C15	1.376 (2)
C3—H3	0.9500	C15—H15	0.9500
C3—C4	1.388 (2)	C15—C16	1.3903 (19)
C4—H4	0.9500	C16—H16	0.9500
C4—C5	1.3813 (17)	C16—C17	1.3804 (17)
C5—C6	1.4044 (16)	C17—C18	1.4035 (16)
C5—C9	1.4983 (17)	C17—C21	1.5006 (16)
C6—C7	1.4643 (16)	C18—C19	1.4639 (15)
C7—C8	1.3402 (17)	C19—C20	1.3434 (16)
C7—C10	1.5032 (16)	C19—C22	1.5051 (15)
C8—C9	1.5051 (16)	C20—C21	1.5065 (16)
C8—C12	1.4947 (17)	C20—C24	1.4916 (17)
C9—H9A	0.9900	C21—H21A	0.9900
C9—H9B	0.9900	C21—H21B	0.9900
C10—H10A	0.9900	C22—H22A	0.9900
C10—H10B	0.9900	C22—H22B	0.9900
C10—C11	1.4654 (17)	C22—C23	1.4646 (17)
C12—H12A	0.9800	C24—H24A	0.9800
C12—H12B	0.9800	C24—H24B	0.9800
C12—H12C	0.9800	C24—H24C	0.9800
			
C2—C1—H1	121.8	C14—C13—H13	121.9
C2—C1—C6	116.30 (12)	C14—C13—C18	116.15 (12)
C6—C1—H1	121.8	C18—C13—H13	121.9
F1—C2—C1	117.80 (13)	F2—C14—C13	117.48 (13)
F1—C2—C3	117.96 (12)	F2—C14—C15	118.12 (12)
C3—C2—C1	124.24 (12)	C15—C14—C13	124.40 (12)
C2—C3—H3	120.6	C14—C15—H15	120.7
C2—C3—C4	118.76 (12)	C14—C15—C16	118.66 (12)
C4—C3—H3	120.6	C16—C15—H15	120.7
C3—C4—H4	120.4	C15—C16—H16	120.5
C5—C4—C3	119.23 (12)	C17—C16—C15	119.08 (12)
C5—C4—H4	120.4	C17—C16—H16	120.5
C4—C5—C6	120.50 (12)	C16—C17—C18	120.72 (11)
C4—C5—C9	131.01 (11)	C16—C17—C21	130.98 (11)
C6—C5—C9	108.49 (10)	C18—C17—C21	108.30 (10)
C1—C6—C5	120.97 (11)	C13—C18—C17	120.99 (11)
C1—C6—C7	131.10 (11)	C13—C18—C19	130.62 (11)
C5—C6—C7	107.93 (10)	C17—C18—C19	108.40 (10)
C6—C7—C10	123.20 (11)	C18—C19—C22	123.15 (10)
C8—C7—C6	110.16 (10)	C20—C19—C18	109.75 (10)
C8—C7—C10	126.63 (11)	C20—C19—C22	127.09 (10)
C7—C8—C9	109.85 (10)	C19—C20—C21	110.04 (10)
C7—C8—C12	128.96 (12)	C19—C20—C24	128.72 (11)
C12—C8—C9	121.19 (11)	C24—C20—C21	121.24 (10)
C5—C9—C8	103.57 (9)	C17—C21—C20	103.51 (9)
C5—C9—H9A	111.0	C17—C21—H21A	111.1
C5—C9—H9B	111.0	C17—C21—H21B	111.1
C8—C9—H9A	111.0	C20—C21—H21A	111.1
C8—C9—H9B	111.0	C20—C21—H21B	111.1
H9A—C9—H9B	109.0	H21A—C21—H21B	109.0
C7—C10—H10A	109.1	C19—C22—H22A	109.1
C7—C10—H10B	109.1	C19—C22—H22B	109.1
H10A—C10—H10B	107.8	H22A—C22—H22B	107.8
C11—C10—C7	112.47 (10)	C23—C22—C19	112.65 (10)
C11—C10—H10A	109.1	C23—C22—H22A	109.1
C11—C10—H10B	109.1	C23—C22—H22B	109.1
N1—C11—C10	179.06 (14)	N2—C23—C22	179.90 (19)
C8—C12—H12A	109.5	C20—C24—H24A	109.5
C8—C12—H12B	109.5	C20—C24—H24B	109.5
C8—C12—H12C	109.5	C20—C24—H24C	109.5
H12A—C12—H12B	109.5	H24A—C24—H24B	109.5
H12A—C12—H12C	109.5	H24A—C24—H24C	109.5
H12B—C12—H12C	109.5	H24B—C24—H24C	109.5
			
F1—C2—C3—C4	−179.90 (11)	F2—C14—C15—C16	−179.92 (12)
C1—C2—C3—C4	−0.3 (2)	C13—C14—C15—C16	0.0 (2)
C1—C6—C7—C8	179.91 (12)	C13—C18—C19—C20	−179.93 (12)
C1—C6—C7—C10	−1.04 (19)	C13—C18—C19—C22	1.14 (18)
C2—C1—C6—C5	−0.34 (17)	C14—C13—C18—C17	0.04 (17)
C2—C1—C6—C7	179.76 (11)	C14—C13—C18—C19	179.88 (11)
C2—C3—C4—C5	0.22 (19)	C14—C15—C16—C17	0.2 (2)
C3—C4—C5—C6	−0.25 (18)	C15—C16—C17—C18	−0.24 (18)
C3—C4—C5—C9	−179.87 (12)	C15—C16—C17—C21	179.58 (12)
C4—C5—C6—C1	0.32 (17)	C16—C17—C18—C13	0.13 (17)
C4—C5—C6—C7	−179.76 (10)	C16—C17—C18—C19	−179.75 (10)
C4—C5—C9—C8	179.75 (12)	C16—C17—C21—C20	179.62 (12)
C5—C6—C7—C8	0.00 (13)	C17—C18—C19—C20	−0.07 (13)
C5—C6—C7—C10	179.05 (10)	C17—C18—C19—C22	−179.00 (10)
C6—C1—C2—F1	179.96 (11)	C18—C13—C14—F2	179.80 (11)
C6—C1—C2—C3	0.3 (2)	C18—C13—C14—C15	−0.1 (2)
C6—C5—C9—C8	0.10 (12)	C18—C17—C21—C20	−0.54 (12)
C6—C7—C8—C9	0.06 (13)	C18—C19—C20—C21	−0.29 (13)
C6—C7—C8—C12	179.99 (12)	C18—C19—C20—C24	179.74 (11)
C6—C7—C10—C11	64.09 (16)	C18—C19—C22—C23	−64.72 (14)
C7—C8—C9—C5	−0.10 (13)	C19—C20—C21—C17	0.51 (12)
C8—C7—C10—C11	−117.02 (13)	C20—C19—C22—C23	116.54 (13)
C9—C5—C6—C1	−179.98 (10)	C21—C17—C18—C13	−179.73 (10)
C9—C5—C6—C7	−0.06 (12)	C21—C17—C18—C19	0.40 (12)
C10—C7—C8—C9	−178.95 (11)	C22—C19—C20—C21	178.59 (11)
C10—C7—C8—C12	1.0 (2)	C22—C19—C20—C24	−1.4 (2)
C12—C8—C9—C5	179.97 (11)	C24—C20—C21—C17	−179.52 (10)

### 2-(5-Fluoro-2-methyl-1H-inden-3-yl)acetonitrile Hydrogen-bond geometry (Å, º)

**Table:**

*D*—H···*A*	*D*—H	H···*A*	*D*···*A*	*D*—H···*A*
C10—H10*B*···N2^i^	0.99	2.65	3.3978 (18)	132
C22—H22*A*···N1^ii^	0.99	2.61	3.4010 (16)	137
C24—H24*C*···F2^iii^	0.98	2.76	3.227 (2)	110
C24—H24*B*···F2^iii^	0.98	2.83	3.227 (2)	105

Symmetry codes: (i) −*x*+1/2, *y*−1/2, −*z*+1/2; (ii) −*x*+3/2, *y*+1/2, −*z*+1/2; (iii) −*x*+3/2, *y*−1/2, −*z*+1/2.
